# Application of morbid animal model in drug safety evaluation of traditional Chinese medicine

**DOI:** 10.3389/fphar.2015.00037

**Published:** 2015-02-27

**Authors:** Ning Ke-Yong, Li Min

**Affiliations:** ^1^Center for Drug Evaluation, China Food and Drug Administration, Beijing, China; ^2^National Shanghai Center for New Drug Safety Evaluation Research Center, Shanghai, China

**Keywords:** TCM, safety evaluation, morbid animal model, pre-clinical, drug

## Abstract

During thousands of years of clinical validation and practice, the efficacy and safety of traditional Chinese medicine (TCM) have been widely recognized in the prevention and treatment for various diseases. However, further toxicology research of TCM new drugs is still necessary.

## NECESSITY OF TOXICOLOGY RESEARCH IN DRUG SAFETY EVALUATION OF TCM

It is well known that many valuable safety information of TCM comes directly from clinical use, which can be perceived as more important reference for TCM safety evaluation, to some extent, even than that from animals. However, the value of previously obtained clinical information will be weakened for various reasons in the development of novel drugs of TCM.

Firstly, the scale of patients treated by TCMs has changed from individuals to populations. Generally, individualized medication was historically used in TCM, with sporadic and individual features. The scientific validity of conclusions from traditional medicine experience is easily to be oppugned due to the individuation of clinical data in traditional medicine experience. Therefore, the toxicology research is urgently needed to support the safe and wide usage of TCM.

Secondly, the awareness of TCM safety has been restricted by the development of subjects and science of that time. In the history of the TCM practice, clinical toxicity observations were based on the development of technology and science at that time. In recent years, some new toxic ingredients and effects in TCM have been recognized, such as the renal toxicity of aristolochic acid in *Caulis aristolochiae* manshuriensis (Chen et al., 2006) and the hepatotoxicity of monocrotaline in *Gynura segetum* (Lin et al., 2011).

Thirdly, some factors may affect the ingredients of novel drugs of TCM compared with that applied historically. Compared with the previous clinical application, the changes of medicinal material source, the prescription composition, extraction technology, selected population, administration mode, dosage, excipients, can lead to functional changes of drug ingredients, which may affect the safety of novel drugs.

Above all, in order to explore the safety risk of TCM, there is great necessity of toxicology research in drug safety evaluation of TCM.

## DEFICIENCIES OF STANDARD ANIMAL MODEL AND NECESSITIES OF MORBID ANIMAL MODEL IN DRUG SAFETY EVALUATION

To evaluate drug safety, there are several deficiencies in current standard animal models, and we should pay more attention to morbid animal models.

### NEED FOR DISEASE ANIMAL MODELS

As is known, the drug is usually used in patients with a morbid status. The ultimate goal of drug safety evaluation is to reduce the safety risk of clinical medication, rather than experimental animals. So there are some deficiencies to predict the safety of drugs in patients with the data generated from normal animals. Adverse reactions might happen in normal animals, but might not occur in patients with the morbid status; and vice versa. What’s more, the exposure of drug in humans cannot solve each problem. Usually we can predict some changes of toxicity in humans with the changes of exposure. But the same exposure may bring different toxicity in humans in different healthy statuses. This different presentation of toxicity may be brought by the different cellular function or different receptor level in healthy people or patients. So in order to predict the clinical toxicity of drugs more accurately, can we evaluate with morbid animal model?

In the theory of TCM, the difference of drug safety revealed in healthy and morbid subjects has been already recognized. The abstruse theory—“You Gu Wu Yun” (有故无殒) means that if a pregnant woman has an abdominal mass, the violent medicine will suit the disease status, both the mother and the fetus will be unharmed. That means, a drug will reveal its therapeutic target when it is prescribed to patients with the right indications, while it may produce deleterious effects in both sick and healthy people as a result of incorrect indications. For example, the blood-activation Chinese medicine can induce miscarriage, but for pregnant women of blood stasis syndrome, it will be less risky.

### NEED FOR CLINICAL TRIALS

Because of the different presentations of toxicity in healthy people or patients, the risk for drugs administrated to patients will increase with the absence of safety data from morbid subjects, including animals or humans. Suppose a drug has not been observed hepatotoxicity in normal animals, it can still cause irreversible liver injury or serious adverse events while being used in patients with decreased liver function or combined with a certain drug with hepatotoxicity.

What’s more, for some special indications, such as tumors, drugs with severe toxicity should not be tested in healthy volunteers with the perspective of ethics. Under this circumstance, patients are often directly used in Phase I clinical trials. Then patients will take a greater risk because of the absence of safety data from the morbid animals as a reference.

### LITERATURE SUPPORTS FOR DISEASE ANIMAL MODELS

There are limitations in predicting drug clinical risk merely by standard animal data. Olson et al. (2000) investigation of the multinational pharmaceutical companies showed that the non-clinical safety study using both rodents and non-rodents can only achieve 71% of consistent prediction with clinically occurred toxicity; while tests only using non-rodents or rodents can achieve 63 or 43% of consistency, respectively. In addition to the species differences between animals and humans, the difference of pathological /physiological statuses may also contribute, at least in part, to the inconsistency.

Some scholars compared toxicity of the same drug between the morbid models and normal animals. Wang et al. (2011) compared toxic effects of rhubarb (with main components that can cause liver damage such as emodin and rhein), a common TCM for treatment of hepatitis, between normal rats and CCl_4_ hepatic injury model rats. Wang found that toxic tolerance of CCl_4_hepatic injury model rats against rhubarb was higher than normal animals. Fang et al. (2011) discovered that concentrations of rhein, emodin and aloe emodin (the main toxic component) in the liver, kidney, spleen of the liver-injured rats was significantly lower than those of normal animals after taken rhubarb extraction orally for 12 weeks. Jiang et al. (2012) found that there were obvious differences in t_1/2_, AUC_0–t_, AUC_0–∞_, T_max_, and CLz/F of paeoniflorin and albiflorin between the normal rats and α-isothiocyanate naphthalene ester (ANIT) induced acute cholestasis hepatitis model rats taken red paeony root, and pharmacokinetics process could be changed in rats with acute liver injury. Based on the above data, drug toxicity and pharmacokinetic process between morbid animals and normal animals tend to be different, which further prompts that adopting the morbid animal model is necessary to safety evaluation.

## CONSIDERATION ON THE MORBID ANIMAL MODEL IN TCM STUDY

Due to the particularity of TCM and the deficiencies of standard animal model in drug safety evaluation, it is necessary to evaluate the non-clinical safety of TCM new drugs by a combination strategy of the standard toxicology model and the morbid animal model.

There are several good reasons. Firstly, the compositions of TCMs are complex and their toxic ingredients are often unclear. Therefore, targeted pharmacokinetic study (ADME/T) cannot be conducted easily, which restricts bridging studies of the safety data from the health status to the pathological status. Secondly, the ADME study of TCM is usually comparatively weak, so that it is difficult to predict the safety of TCM in patients with ADME data. Thirdly, there are deficiencies of standard animal models in drug safety evaluation. So sometimes we need to consider the use of morbid animal models in safety study of TCM.

## RELATED CONTENT IN GUIDELINES

Although there is no specific guideline using morbid animal models in non-clinical drug safety evaluation, some guiding principles have referred to this in International Conference on Harmonization (ICH).

In ICH, 2011, it has been pointed out that the alternative study by using animal models similar to human disease could be used in drug safety evaluation: these animal models include spontaneous or induced animal models of disease, gene knockout and transgenic animals. These models may provide further insight, not only in determining the pharmacological action of the product, pharmacokinetics, and dosimetry, but may also be useful in the determination of safety (e.g., evaluation of undesirable promotion of disease progression). In certain cases, studies performed in animal models of disease may be used as acceptable alternative models to toxicity studies in normal animals.

Although such models are referred to biological drugs in ICH S6, universal significance should be considered to all kinds of drugs including TCM.

In ICH, 2005, it has also been mentioned that animal models might have utility in assessing proarrhythmia of drugs.

In ICH, 2009, the application of the morbid animal model was not mentioned in non-clinical safety evaluation of anticancer drugs. But in order to predict the safety of the drug in cancer patients more accurately, there are still a part of applicants who adopted tumor-burdened animals for non-clinical safety evaluation in the development of anti-cancer drugs (Ferreira et al., 2012).

## PERSPECTIVE

In recent years, researches on disease mechanism, basic sciences and morbid animal models have progressed rapidly. That made it easier for us to study the safety of drugs with morbid animals.

On the other hand, as with standard animal models, adopting morbid animal models also has limitations in predicting drug safety to the patients. Due to the short development time and limited basic research, adopting morbid animal models in non-clinical safety study may face certain challenges such as limited historical background data and difficulties in interpreting study data. So it is essential to collect control data and baseline data in the same period in order to draw more scientific conclusions.

In spite of some deficiencies, in order to predict the clinical toxicity of drugs as accurately as possible and reduce the risks to clinical subjects, it is necessary to pay more attention to the use of morbid animals in pre-clinical safety research. Because of the characteristics of TCM, safety studies should also be considered in research and development of new drugs of TCM, and sometimes using morbid animal models could be a good choice. For some new drugs that include high risk Chinese medicines (e.g., that with narrow safety margin, severe toxicity, steep dose-response, prone to accumulation, etc.), or other conditions that using for high-risk groups or health factors that may influence drug reaction, morbid animal models could be used as the necessity for non-clinical safety evaluation.

### Conflict of Interest Statement

The authors declare that the research was conducted in the absence of any commercial or financial relationships that could be construed as a potential conflict of interest.
